# Intraventricular Subependymoma With Obstructive Hydrocephalus: A Case Report and Literature Review

**DOI:** 10.7759/cureus.52563

**Published:** 2024-01-19

**Authors:** Corneliu Toader, Razvan-Adrian Covache-Busuioc, Bogdan-Gabriel Bratu, Luca-Andrei Glavan, Andrei Adrian Popa, Matei Serban, Alexandru Vladimir Ciurea

**Affiliations:** 1 Department of Neurosurgery, Carol Davila University of Medicine and Pharmacy, Bucharest, ROU; 2 Department of Neurosurgery, National Institute of Neurology and Neurovascular Diseases, Bucharest, ROU; 3 Department of Neurosurgery, Sanador Clinical Hospital, Bucharest, ROU

**Keywords:** gross total resection, obstructive hydrocephalus, tumor resection, subependymoma, intraventricular tumor

## Abstract

Subependymomas are benign tumors of the ventricles that grow from the ventricular wall into the cerebrospinal fluid spaces within the brain, obstructing the flow of the cerebrospinal fluid and causing obstructive hydrocephalus. It is estimated that ependymomas represent between 0.2% and 0.7% of all intracranial tumors. They arise most frequently in the fourth ventricle (50-60%) and the lateral ventricles (30-40%). We present the case of a 50-year-old patient, previously diagnosed with an intraventricular process, admitted in our clinic. At neurological examination, the patient was cooperative, bradylalic, and bradypsychic, with right hemiparesis, postural and balance disorders, and occasionally sphincteric incontinence. MRI with contrast described a left intraventricular tumor, in the frontal horn of the left lateral ventricle with homogeneous appearance, with a maximum diameter of 50 mm and base of insertion at the adjacent ependyma of the foramen of Monro, which determined obstructive hydrocephalus. Total resection of the left intraventricular cerebral tumor was achieved. Histopathological analysis revealed a subependymoma. Postoperative recovery was slowly favorable, with significant neurological improvement. At neurological examination at three-month follow-up, the patient's right hemiparesis and unsystematized balance disorders improved. A contrast-enhanced CT scan was performed, highlighting left frontal sequelae hypodensity corresponding to the operated tumor, enlarged left lateral ventricle without active hydrocephalus, and no sign of tumor recurrence. At six-month follow-up, clinico-radiologic findings coincide with those from three-month follow-up. Subependymomas are slow-growing (grade 1) tumors and generally have a favorable prognosis. Unfortunately, due to their anatomical level, multiple complications can arise, caused from obstructive hydrocephalus complications, such as cognitive dysfunction and incontinence. Tumor resection should be complete, a successful operation being a challenge for every neurosurgeon.

## Introduction

Subependymomas (SE) are uncommon, slow-growing neoplasms, histologically categorized as low grade (World Health Organization (WHO) grade 1) [1]. They account for only 0.2-0.7% of all central nervous system tumors [2,3]. SE can affect individuals of any age or sex, but they predominantly arise in middle-aged to elderly people, often in their 50s and 60s [4]. While most cases are asymptomatic, symptoms can manifest in larger tumors located in areas like the lateral, third, and fourth ventricles, the septum pellucidum, and occasionally the spinal cord, primarily due to hydrocephalus or spontaneous tumor hemorrhage [5]. Despite their generally benign nature, there have been reports of tumor recurrence and central nervous system metastasis, commonly in cases where a subtotal resection was undergone [6].

SE emerge in various parts of the central nervous system, predominantly originating from the fourth and lateral ventricles. Histologically, these tumors are characterized as circumscribed gliomas with negligible or absent mitotic activity and a lack of pronounced nuclear atypia. In cases where lesions are not clearly defined, a DNA methylation profile consistent with supratentorial SE (ST SE), posterior fossa SE (PF SE), or spinal SE (SP SE) is necessary. Clinically, SE are often asymptomatic and primarily present in adults, with a median onset age around 50 years [7]. The overall prognosis for SE is favorable, and the occurrence of recurrence is exceedingly rare, even in cases of subtotal resection. However, SE located in the PF tend to exhibit a marginally more aggressive course, with a five-year progression-free survival (PFS) rate of 83%, in contrast to a 100% PFS rate in ST and SP SE.

SE are often discovered incidentally during autopsies. However, symptomatic cases do occur. In these instances, patients typically exhibit symptoms associated with cerebrospinal fluid obstruction or mass effect due to the tumor's presence [8].

The scarcity of SE cases has led to a lack of definitive treatment guidelines. For symptomatic SE, complete surgical excision is commonly recommended [9]. However, achieving this can be challenging, especially for tumors located in the fourth ventricle, where there is proximity to the brainstem and cranial nerves. In some cases, staged surgery or subtotal resection has been shown to yield favorable outcomes [10]. Radiation therapy has been employed following subtotal resection, but its precise role remains uncertain due to limited application [11].

## Case presentation

A 50-year-old patient recently diagnosed, following gait and balance disorders, with intraventricular intracranial expansive process was admitted to our service for specialized therapeutic management. At neurological examination on admission, the patient was conscious, cooperative, bradylalic, and bradypsychic, with right hemiparesis, gait and orthostasis disorders, unsystematized balance disorders, and sphincter content disorders. Moreover, psychiatric assessment was undergone considering the anxious behavior of the patient, but nothing notable was observed.

**Figure 1:**
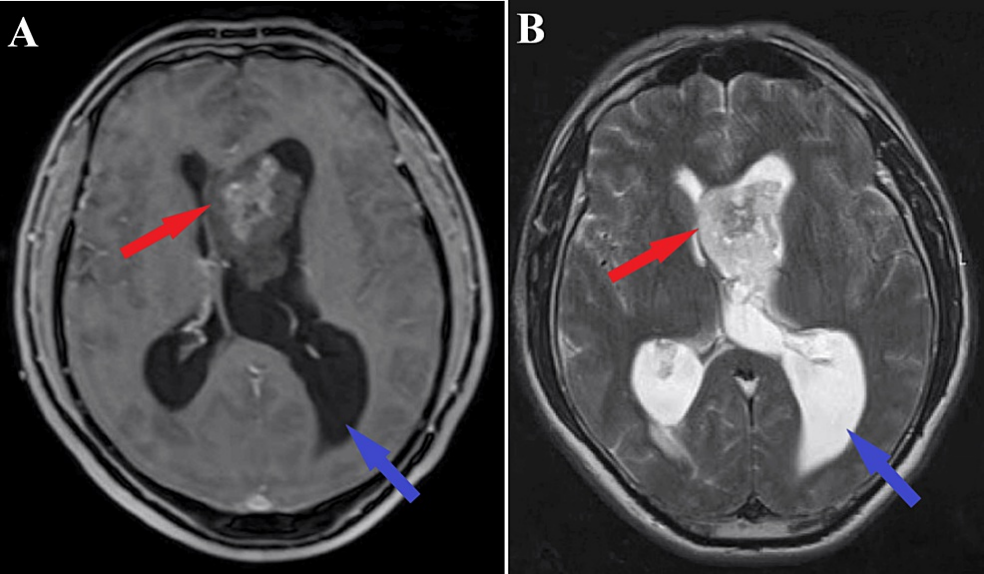
Preoperative T1Gd and T2 sequence MRI The axial section of MRI T1 sequence with gadolinium (A) and axial section of MRI T2 sequence (B) depict the intraventricular tumoral process (red arrows) with the associated significant obstructive hydrocephalus (blue arrows)

The brain MRI scan showed a voluminous left intraventricular intracranial expansive process (frontal horn of the left lateral ventricle), homogeneous in appearance, hypointense in T1, hyperintense in T2, with a maximum diameter of about 50 mm and base of insertion at the adjacent ependyma of the foramen of Monro, causing obstructive internal hydrocephalus (Figure 1).

**Figure 2:**
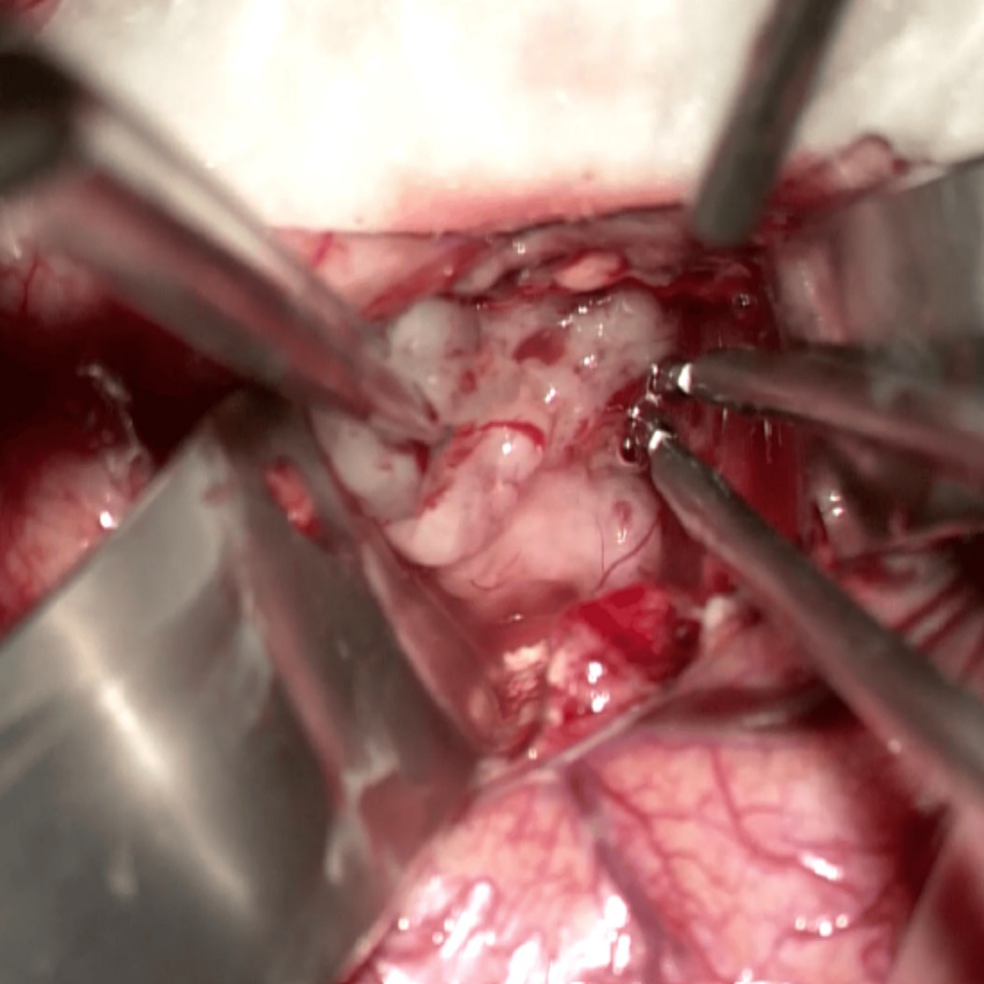
Intraoperative image of tumoral mass The image shows the intraoperative aspect of intraventricular tumor as a whitish-gray tumor; bipolar cauterization can be seen used for vascularity within the tumoral mass

During surgery, an anterior transcortical approach through the middle frontal gyrus was performed, being the appropriate approach for patients with hydrocephalus. Under the operating microscope, at the frontal horn of the left lateral ventricle, a well-marked whitish-gray tumor was exposed (Figure 2).

**Figure 3:**
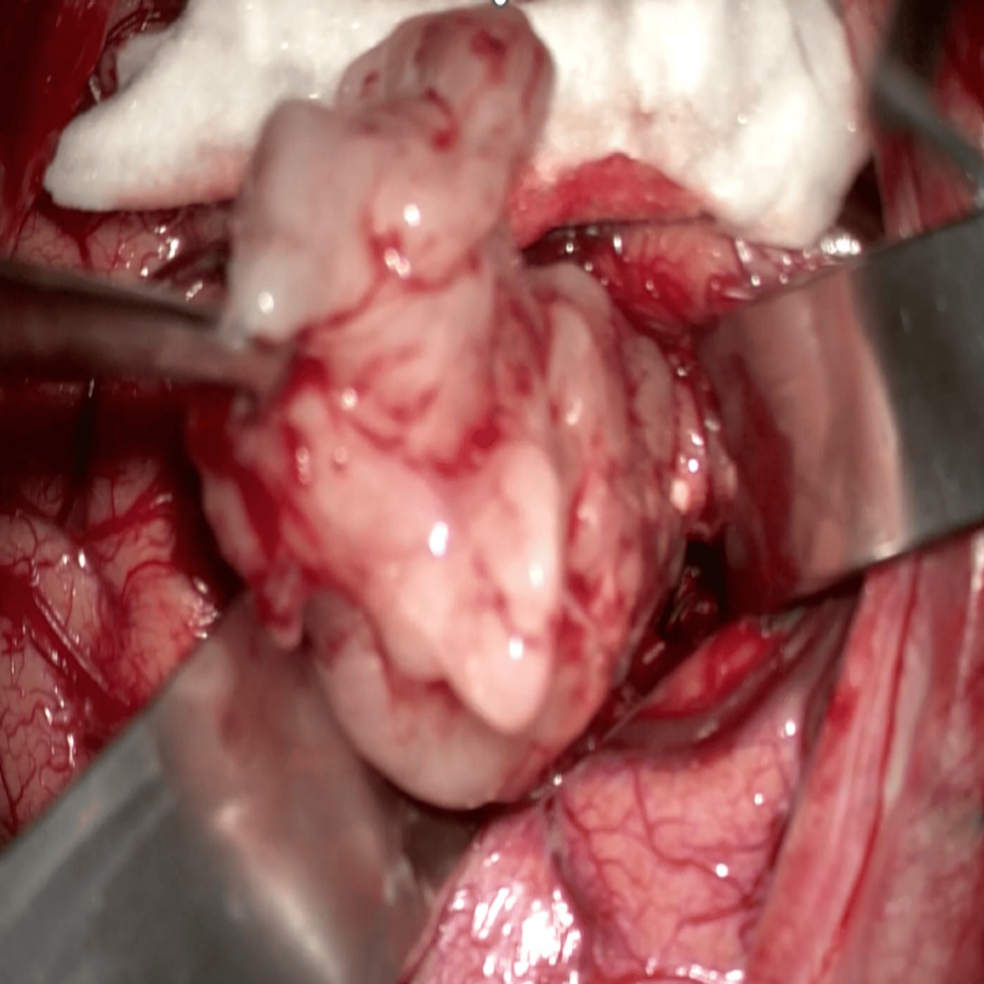
Intraoperative image Gross tumor resection was achieved; the image depicts the total removal of tumoral process

From the beginning of the dissection, fragments were collected for histopathological examination. Coagulation and incision of the tumor capsule were performed, maintaining the integrity of the fornix, caudate nucleus, thalamus, and surrounding vasculature for minimal postoperative neurological deficits. Total tumor resection was successful (Figure 3), and after the mass was removed, the ventricular surface was inspected for any adherent tumor fragments. Histopathological examination confirmed the diagnosis of SE.

**Figure 4:**
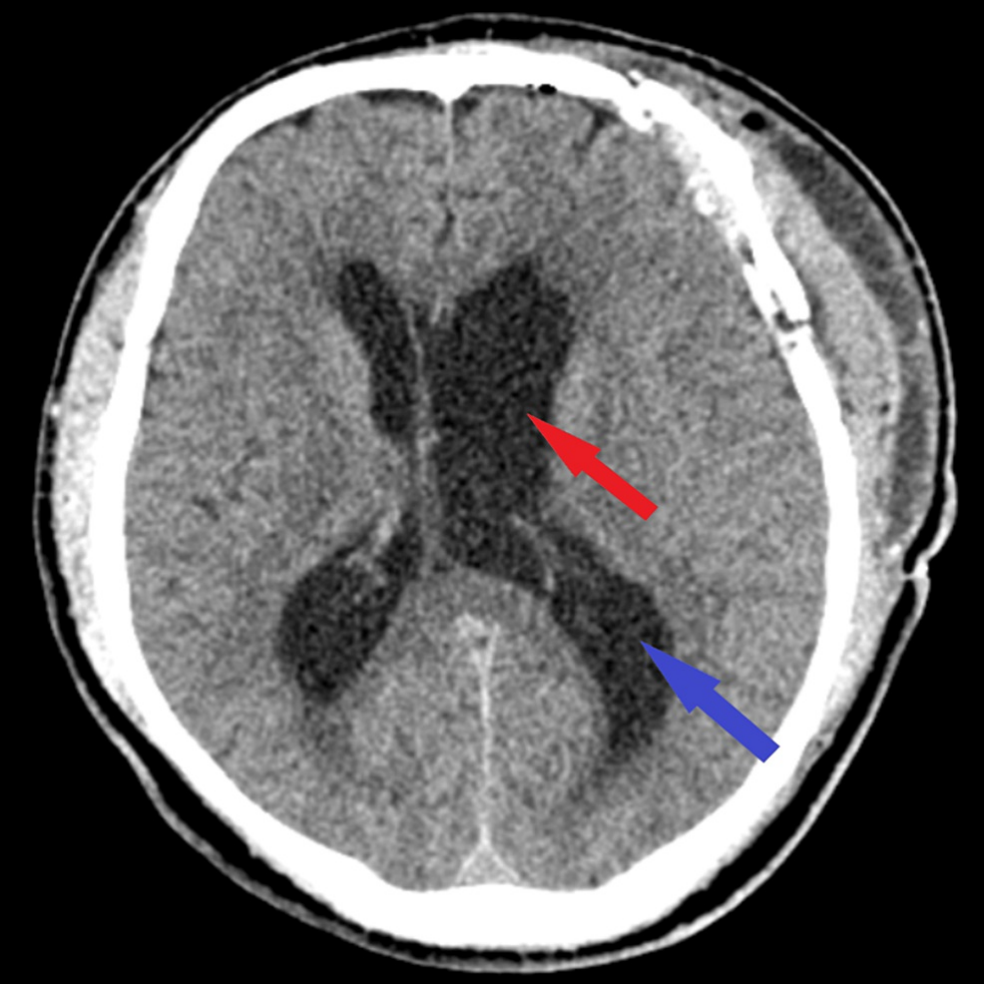
Postoperative CT scan The axial section of the CT scan performed postoperatively highlights total tumor resection (red arrow) as well as an enlarged left lateral ventricle (blue arrow)

Postoperatively, the patient presented a normal evolution, with significant neurological improvement; on CT scan, an enlarged left lateral ventricle without active hydrocephalus and no sign of tumor recurrence was seen (Figure 4). At discharge, the patient was conscious, cooperative, and coherent, with remitted right hemiparesis, remitted orthostasis disorders, and spontaneous diuresis.

**Figure 5:**
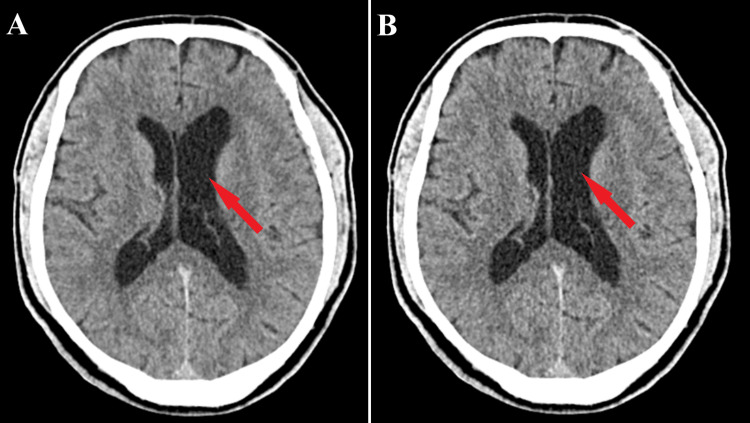
Follow-up CT scan The axial section of the CT scan at three-month follow-up (A) and axial section of the CT scan at six-month follow-up (B) both show no residual tumor or recurrence (red arrows) with a slightly enlarged left lateral ventricle

At three-month follow-up, the patient was conscious and coherent, without motor deficits, but balance disturbances and memory and attention disturbances were present. There was a slight favorable improvement, neurologically almost fully recovered. At the next check-up after six months, the patient was conscious and coherent, and unsystematic balance disturbances, memory and attention disturbances, right-sided tactile hemiparesis, and occasional sphincter incontinence were present. The patient has slow favorable improvement and small neurological improvements. In both control imaging, the CT aspect revealed a left frontal sequelae hypodensity corresponding to the operated tumor, an enlarged left lateral ventricle without active hydrocephalus, and no sign of tumor recurrence (Figure 5).

## Discussion

SE expand slowly and non-invasively. The majority of these tumors remain asymptomatic and are typically less than 2 cm in size [12]. However, it is not unusual to find tumors exceeding 4 cm, which often become symptomatic due to obstructive hydrocephalus. In such cases, patients may exhibit symptoms indicative of intracranial hypertension, including headache, dizziness, nausea, or cerebellar signs like gait ataxia. Additionally, cognitive decline, seizures, and paresis have been observed in some patients.

In a retrospective analysis of 13 patients with intracranial WHO grade 1 SE from 1990 to 2015, Varma et al. demonstrated that some intraventricular SE could be managed conservatively with MRI monitoring. The study found no significant disease progression during an average follow-up of 46 months, suggesting that long-term follow-up might not be necessary [13]. Additionally, it was noted that hydrocephalus was the primary complication in the surgical treatment of hydrocephalic SE [14], reinforcing the viability of conservative treatment in certain cases. This approach may seem counterintuitive given the low malignancy rate of SE and the rarity of deaths directly caused by the tumor in a short period. Often, patients are more likely to succumb to accidents or other factors.

For symptomatic SE, the recommended treatment is complete surgical excision [15]. However, partial resection can also yield favorable outcomes and is an acceptable option, particularly when tumors are located near critical areas where surgery might pose risks. The primary surgical objective should be the restoration of normal cerebrospinal fluid flow. Historically, perioperative mortality rates were as high as 28.8% before the advent of microsurgical techniques. Currently, with advancements in technology and neurosurgical methods, such high mortality rates are largely anecdotal, and surgery-related complications are rare [16,17].

Immunotherapeutic strategies offer promising benefits for treating SE due to their minimal invasiveness and negligible cytotoxic effects. The overexpression of specific antigens in glioma cells, identified as potential therapeutic targets, has led to the development of vaccines targeting these antigens. Proteins such as survivin, EphA, IL-13RA, EGFRvIII, CMV antigens, and nucleolin are known to be overexpressed in gliomas, including low-grade gliomas [18,19], suggesting they could be potential targets for SE immunotherapy. Peptide and aptamer vaccines targeting these antigens are currently available or in development [20].

Another avenue for treatment is the blockade of operational immunosuppressive pathways in SE. Several agents targeting these tumor-mediated immunosuppressive pathways, such as CTLA-4, Tregs, and PD-1, are either available or under clinical investigation [21,22]. To determine the suitability of immunotherapy for SE, it is essential to first understand the antigenic profile and immunosuppressive mechanisms within the tumor. Additionally, key signaling pathways commonly upregulated in gliomas, such as the signal transducer and activator of transcription 3 (STAT3), the epidermal growth factor (EGF), and the platelet-derived growth factor (PDGF), present potential therapeutic targets.

Intraventricular ependymomas typically grow relatively slowly and often remain asymptomatic until they reach a considerable size at diagnosis. Common clinical manifestations include cognitive decline and symptoms of intracranial hypertension resulting from impaired cerebrospinal fluid circulation and consequent hydrocephalus. The most frequently reported symptoms in various studies are related to intracranial hypertension (like headache and papilledema), and imaging often shows obturative hydrocephalus. Less common signs and symptoms in patients with intraventricular tumors are seizures, focal deficits, and cerebellar ataxia in PF cases. About 8% of tumors located in the lateral ventricle or trigonum present with focal deficits. The treatment protocol for intracranial ependymomas includes surgical resection followed by postoperative radiotherapy [23]. The surgical approach varies based on the primary site of tumor expansion, extent of intraventricular growth, transependymal spread into the periventricular zone, and tumor size. We delved into multiple studies focused on intraventricular SE surgical treatment and mentioned the possibility of recurrence and hydrocephalus for a wide point of view regarding complete tumor resection and associated postoperative complications (Table 1).

**Table 1:** Overview of intraventricular subependymoma cases in the current literature M: male; F: female; GTR: gross tumor resection; NA: not available

Author/year	Case no.	Age (years)	Sex ratio	GTR	Recurrence	Hydrocephalus post-op	Mean follow-up (years)
Landriel et al. 2013 [24]	1	33	M	100%	0%	0%	1
Hernández‑Durán et al. 2014 [25]	1	51	M	100%	0%	0%	1
Agarwal et al. 2014 [26]	1	44	F	100%	0%	100%	NA
Tiwari et al. 2015 [27]	1	62	F	100%	100%	0%	6
Nguyen et al. 2017 [28]	466	Mean age=40	F:M=3:7	35.1%	NA	15%	3.5
D'Amico et al. 2017 [29]	26	Mean age=57	F:M=4:6	95%	0%	NA	2.9
Hanashima et al. 2018 [30]	1	69	M	100%	0%	0%	1
Ordones et al. 2019 [31]	2	Mean age=40.5	F:M=2:0	100%	0%	0%	NA
Fuchinoue et al. 2022 [32]	1	81	M	0%	0%	NA	10
Iwanowski et al. 2023 [33]	1	49	M	100%	0%	0%	4

The use of magnetic resonance perfusion (MRP) or magnetic resonance spectroscopy (MRS) in diagnosing SE is not widely reported [34]. In 2012, Abdel-Aal et al. reported a case of an intraventricular SE in the left lateral ventricle, suggesting that hyperperfusion in SE might result from the presence of thick hyalinized vessels, as opposed to the neoangiogenesis seen in high-grade tumors [35]. MRS in low-grade tumors typically shows normal choline (Cho) levels and slightly reduced N-acetylaspartate (NAA) levels. By focusing on these enhancing features, MRS and MRP can be instrumental in providing accurate diagnoses and aiding in the formulation of effective treatment plans.

## Conclusions

Intraventricular ependymomas and SE still remain a surgical challenge due to a relatively high incidence of incomplete tumor resections and/or permanent neurological complications associated with their removal. Still, even incomplete tumor removal with subsequent radiotherapy facilitates long-term PFS in some cases.

In conclusion, these benign tumor formations called SE are rare, and their arrangement and size influence the flow of cerebrospinal fluid leading to obstruction. They have an optimal response to surgery, ideally total resection. In order to achieve the best outcome for the patient, prompt and accurate diagnosis is vital. This pathology needs to be further studied, both to more easily detect or confirm its presence in a patient's body and to further investigate and discover new methods of treatment.
